# Preparation of Anti-Aristolochic Acid I Monoclonal Antibody and Development of Chemiluminescent Immunoassay and Carbon Dot-Based Fluoroimmunoassay for Sensitive Detection of Aristolochic Acid I

**DOI:** 10.3390/foods10112647

**Published:** 2021-11-01

**Authors:** Ai-Fen Ou, Zi-Jian Chen, Yi-Feng Zhang, Qi-Yi He, Zhen-Lin Xu, Su-Qing Zhao

**Affiliations:** 1 Department of Pharmaceutical Engineering, School of Biomedical and Pharmaceutical Sciences, Guangdong University of Technology, Guangzhou 510006, China; ouaifen@gcp.edu.cn (A.-F.O.); chesto36@163.com (Q.-Y.H.); 2 Department of Food, Guangzhou City Polytechnic, Guangzhou 510006, China; 3 Guangdong Provincial Key Laboratory of Food Quality and Safety/Guangdong Laboratory of Lingnan Modern Agriculture, South China Agricultural University, Guangzhou 510642, China; guangdongchenzj@163.com (Z.-J.C.); zhyifengscau@163.com (Y.-F.Z.); jallent@163.com (Z.-L.X.)

**Keywords:** aristolochic acid I, monoclonal antibody, computer-assisted simulation, chemiluminescent immunoassay, fluoroimmunoassay

## Abstract

Aristolochic acid (AA) toxicity has been shown in humans regarding carcinogenesis, nephrotoxicity, and mutagenicity. Monitoring the AA content in drug homologous and healthy foods is necessary for the health of humans. In this study, a monoclonal antibody (mAb) with high sensitivity for aristolochic acid I (AA-I) was prepared. Based on the obtained mAb, a chemiluminescent immunoassay (CLEIA) against AA-I was developed, which showed the 50% decrease in the RLU_max_ (IC_50_) value of 1.8 ng/mL and the limit of detection (LOD) of 0.4 ng/mL. Carbon dots with red emission at 620 nm, namely rCDs, were synthesized and employed in conventional indirect competitive enzyme-linked immunosorbent assay (icELISA) to improve the assay sensitivity of a fluoroimmunoassay (FIA). Oxidized 3,3′′,5,5′′-tetramethylbenzidine dihydrochloride (oxTMB) can quench the emission of the rCDs through the inner-filter effect; therefore, the fluorescence intensity of rCDs can be regulated by the concentration of mAb. As a result, the assay sensitivity of FIA was improved by five-fold compared to CLEIA. A good relationship between the results of the proposed assays and the standard ultra-high performance liquid chromatography-triple quadrupole mass spectrometer (UPLC-QQQ-MS/MS) of real samples indicated good accuracy and practicability of CLEIA and FIA.

## 1. Introduction

Aristolochic acids (AAs) are a mixture of structurally related nitrophenanthrene carboxylic acids, mainly consisting of aristolochic acid I (AA-I) and aristolochic acid II (AA-II), which exist in Aristolochia spp. [1,2], a kind of Chinese herb. Moreover, these herbs can be used as raw materials of some drug homologous and healthy foods, and even dietary supplements [3,4,5]. However, it has been reported that AA showed toxicity to humans owing to carcinogenesis [2,6,7,8], nephrotoxicity [9,10,11], and mutagenicity [9,12]. Some cases reported that the intake of slimming products containing AA resulted in nephropathy [13,14]. Many countries have prohibited products containing AAs. Therefore, it is necessary to develop effective methods for detecting and monitoring AA in related food products.

For the analysis of AA, the main detection method is the conventional instrumental method, high performance liquid chromatography (HPLC) [15,16,17], due to the high accuracy and high reproducibility. Nevertheless, it is a challenge that the instrumental method is limited by high cost, the need for professional operators, and a long turnaround time. Therefore, immunoassay was proposed in this study because of the advantages of rapidness, high-throughput, sensitivity, low-cost, simple pretreatment requirement. It is easy-to-use and has been widely applied in fields of food analysis [18,19,20,21,22,23,24].

The most common and mature detection technology for the immunoassay of AAs is conventional indirect competitive enzyme-linked immunosorbent assay (icELISA); however, the sensitivity of icELISA cannot meet the needs of strict screening. In this study, a chemiluminescent immunoassay (CLEIA) was developed concerning its higher sensitivity compared with conventional icELISAs [25,26]. On the other hand, a fluoroimmunoassay (FIA) is a potential methodology through its advantages, including high sensitivity, real-time, fast response, and low cost to improve the method sensitivity [27]. As a novel fluorescent probe, the carbon dots (CDs) exhibit superiority of remarkable fluorescent properties, simple preparation, good biocompatibility, and easy functionalization [28], which can be employed for FIA. For FIA development, most previous publications reported a phosphate-triggered method to recover the fluorescence of CDs [29,30,31]. Nevertheless, alkaline phosphatase (ALP) activity requires more than 30 min for the catalyst, which is not satisfactory. For overcoming this time-consuming step, we employed horseradish peroxidase (HRP) and developed red CDs (rCDs)-based FIA. Compared with ALP, the activity of HRP is higher and its catalysate-oxidized 3,3′′,5,5′′-tetramethylbenzidine dihydrochloride (oxTMB) can quench rCDs. Based on the above principle, we developed HRP and rCDs based on FIA for AAs analysis. 

## 2. Materials and Methods

### 2.1. Reagents and Animals

Standards of AA-I, AA-II, AA-III, AA-IV, and its analogs were purchased from Yuanye Co. Ltd. (Shanghai, China). Citric acid, urea, N,N-dimethylformamide (DMF), 1-(3-dimethylaminopropyl)-3-ethylcarbodiimide hydrochloride (EDC), N-hydroxysuccinimide (NHS) were supplied by Heowns Chemical Technology Co., Ltd. (Tianjin, China). The ovalbumin (OVA), keyhole limpet haemocyanin (KLH), and bovine serum albumin (BSA) were supplied by Sigma (Shanghai, China). The incomplete and complete Freund’s adjuvants were purchased from Merck Co. Ltd. (Shanghai, China). The TMB and chemiluminescent substrate solution were obtained from Yuanye Co. Ltd. (Shanghai, China). Protein G resin and a secondary antibody (goat anti-mouse IgG, HRP conjugated) were obtained from TransGen Biotech Co. Ltd. (Beijing, China).

Bal b/c female mice were purchased from the Guangdong Medical Experimental Animal Centre and raised at the Animal Experiment Centre of South China Agriculture University (Animal Experiment Ethical Approval Number: 2019054, Figure S1).

### 2.2. Instruments

Multiskan FC microplate reader (Thermo Fisher, Shanghai, China) was used to measure absorbance values. The fluorescence was measured at emission (Em) wavelengths of 620 nm with excitation (Ex) wavelength of 540 nm using a SpectraMax i3 microplate reader (Molecular Devices, USA). A NanoDrop2000c spectrophotometer (Thermo Fisher, Shanghai, China) was used for concentration measurement and UV spectrum characterization.

### 2.3. Production of Monoclonal Antibody

Since AA-I is the main compound of AAs, it was directly conjugated to a carrier protein to prepare immunogen and coating antigen. The conjugation procedure was referred to in our previous study [32], and the details are summarized in Supporting Information. The artificial antigens were characterized by ultraviolet visible (UV-vis) spectral. The molar ratio between hapten and carrier protein was obtained by MALDI-TOF-MS.

The produced artificial antigens were used for animal immunization described in our previous study [32]. The production of mAb was followed by our previous publication [33]. The obtained mAb was purified by protein G and stored at −20 °C.

### 2.4. Development of Chemiluminescent Immunoassay

A serial concentration of AA-I (50 μL) and 50 μL of mAb solution was added to each well for 40 min incubation at 37 °C. Afterward, HRP-conjugated secondary antibody was added (100 μL/well) after five times washing with PBST (PBS with 0.5 ‰ Tween-20) for 30 min incubation at 37 °C. Then the chemiluminescent substrate solution was added (100 μL/well), and the RLU value was measured after a 1 min reaction. The calibration curve was fitted by sigmoidal fitting using the percent binding of mAb in the wells (RLU/RLU_0_) against the logarithm of the AA-I concentration. The 50% decrease in the RLU_max_ (IC_50_) value was calculated using Origin 8.5. The optimal conditions were confirmed from the IC_50_ value, including the optimal pH, coating antigen/antibody, phosphate, and Tween-20 concentration.

### 2.5. Development of Fluoroimmunoassay

The synthesis of rCDs is summarized in Supporting Information for the development of FIA. It was the same as CLEIA except using TMB as substrate and polystyrene transparent microplate. After oxTMB generation, the solution of each well was mixed with 50 μL of NaOH (1 mM, pH 11) to adjust the pH. Then, rCDs (50 μL) were added and mixed quickly. One hundred microliters of the mixture were transferred to black polystyrene opaque microplate, and the fluorescence signal was measured with Ex 540 nm and Em 620 nm.

### 2.6. Recovery Test

Samples (drug homologous and foods) were obtained from a supermarket in Guangzhou city. Samples (600, 300, and 150 ng/g) were ground into a powder using a stainless-steel grinder. The AA-I was added to samples (5.0 g) and mixed with methanol (5 mL) for 30 min ultrasonic water bath treatment. Afterward, the mixture was centrifuged at 4000 rpm (2057× *g*) for 10 min. The extraction solution was dried by nitrogen flow and redissolved in an equivalent volume of 0.01 M pH 5.4 PBS (PB with 75 mM NaCl) and employed for CLEIA and FIA. For UPLC-QQQ-MS/MS, the redissolved solutions were filtered by 0.22 μm cellulose membrane before analysis. The details of UPLC-QQQ-MS/MS are summarized in Table S1.

## 3. Results

### 3.1. Characterization of Antisera

**Figure 1:**
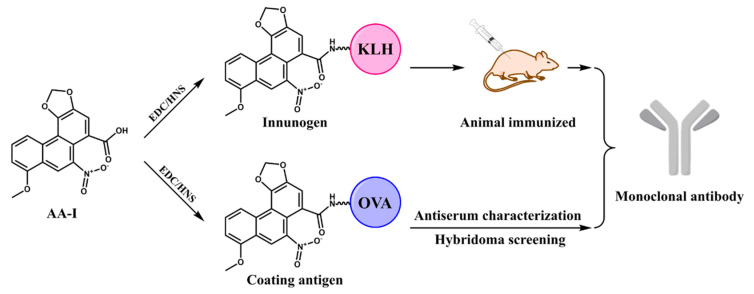
The strategic schema of mAb production. AA-I: aristolochic acid I; EDC: 1-(3-dimethylaminopropyl)-3-ethylcarbodiimide hydrochloride; NHS: N-hydroxysuccinimide; KLH: keyhole limpet haemocyanin; OVA: ovalbumin.

In this study, AA-I was conjugated to a carrier protein directly. The ultraviolet scanning showed that the synthesized artificial antigens exhibited the characteristic absorption peak of AA-I and carrier proteins, suggesting the successful preparation of artificial antigens (Figure S2). The prepared immunogen and coating antigen were further utilized to prepare anti-AA-I mAb. The strategy is shown in Figure 1. The mouse antisera characterization is summarized in Table 1. The highest titer and sensitivity were observed for mouse 4 using AA-I-KLH, which was chosen for the production of mAb. After subclone and hybridoma screening, the most sensitive cell lines 3A3 were obtained and used for ascites preparation (Table S2). Finally, the mAb was obtained after ascites purification and was stored at −20 °C after concentration measurement by Nanodrop.

### 3.2. Development of CLEIA

**Figure 2:**
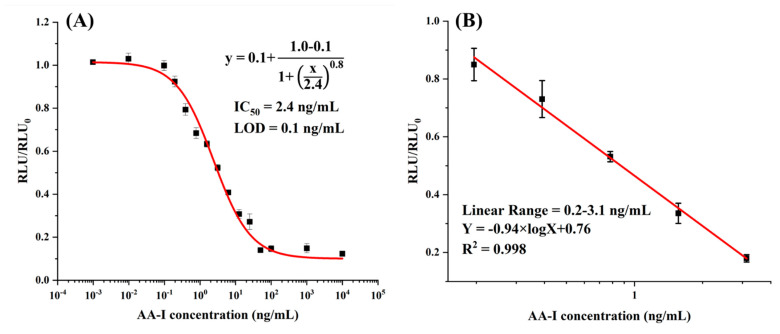
(**A**) The calibration curve; (**B**) the linear range of CLEIA.

After condition optimization, the CLEIA was further developed. The highest sensitivity was achieved with the coating concentration of 2 μg/mL and antibody concentration of 250 ng/mL (Table S3). For the working buffer, the optimized solution was pH 5.4 PBS with a concentration of 0.01 M (Figure S3). Finally, calibration curves against AA-I were developed and showed the IC_50_ of 2.4 ng/mL with a linear range (IC_20_–IC_80_) from 0.2 to 3.1 ng/mL (Figure 2). The IC_10_ (10% decrease in the RLU_max_) was defined as the limit of detection (LOD), 0.4 ng/mL.

Based on the developed CLEIA, the specific test for mAb was performed, and the results are summarized in Table 2. To be noticed, the AA-II showed the highest cross-reactivity (CR) amount of these analogs while only slight CR was observed for AA-III and AA-IV, suggesting the hydroxyl was the key site for recognition. For the other analogs, no obvious CR was observed, indicating the good specificity of mAb to AAs. Since both AA-I and AA-II were the main components in the samples, the obtained mAb can be used for screening these two AAs.

### 3.3. Characterization of rCDs

**Figure 3:**
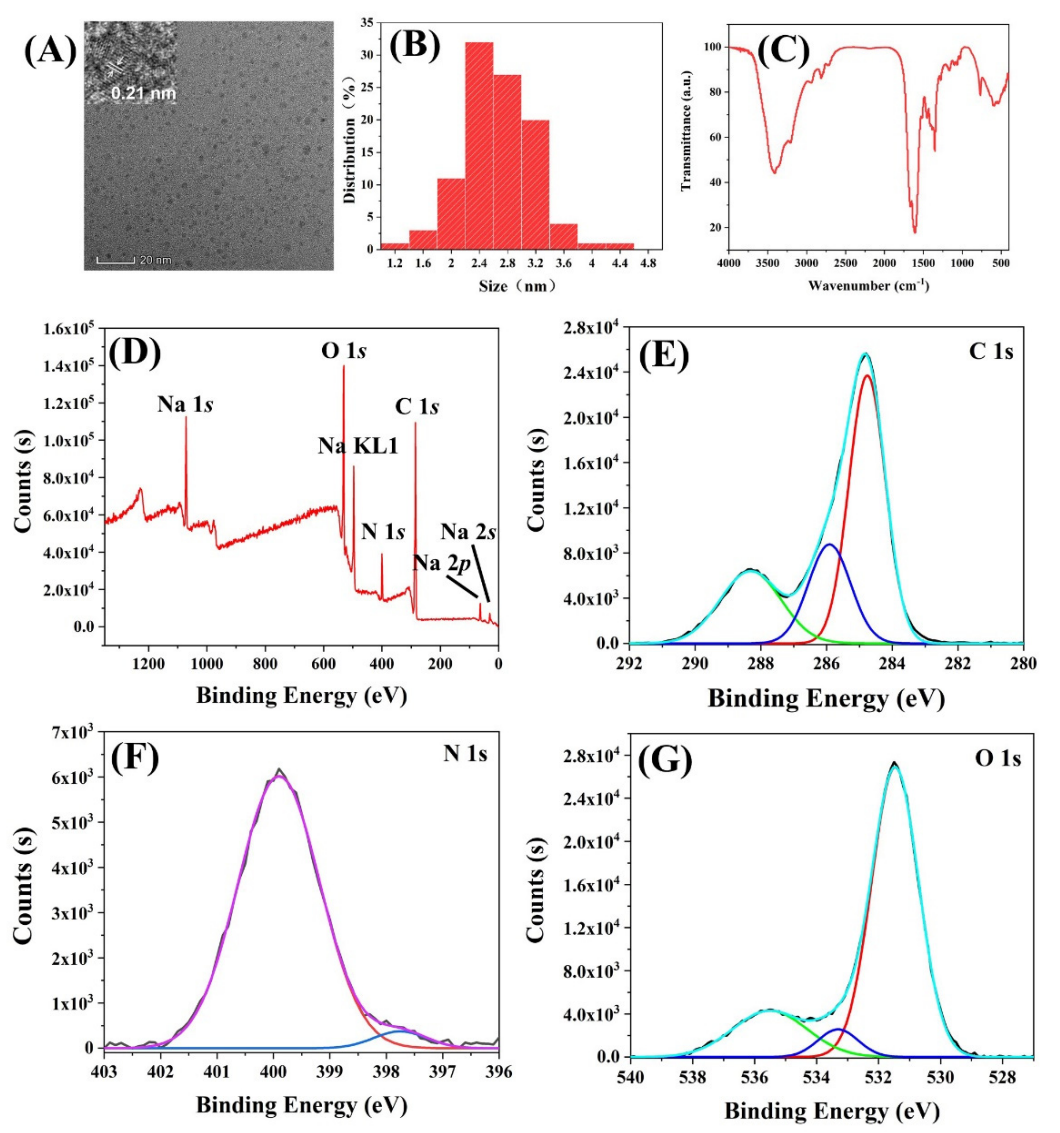
Characterization of rCDs; (**A**) the transmission electron microscope image; (**B**) the hydrodynamic size; (**C**) FTIR image; (**D**) survey XPS spectrum and high resolution of (**E**) C 1*s*, (**F**) N 1*s*, and (**G**) O 1*s*.

The rCDs were synthesized and characterized for the development of FIA. As shown in Figure 3A, rCDs exhibited a particle size of approximately 3 nm. Moreover, the high-resolution TEM image clearly showed the lattice fringe of rCDs with an interlayer spacing of 0.21 nm (Figure 3A). The dynamic light scattering (Figure 3B) showed the hydrodynamic size of ~2.7 nm for rCDs, which agreed with TEM.

The Fourier transform infrared (FTIR) was employed to characterize the functional group of rCDs (Figure 3C). A wide peak from 3100 to 3500 cm^−1^ was found from the FTIR image, which was assigned to the stretching vibrations of the O-H or N-H group, while those from 2800 to 3000 cm^−1^ could be attributed to the stretching vibrations of C-H. The peaks at 1610, 1510, and 1450 cm^−1^ indicated the generation of the aromatic group. The stretching vibrations of C=O or C=N can be verified from the peak of 1680 cm^−1^, and the peak at 1350 cm^−1^ was caused by the stretching vibrations of C-N, which demonstrated the generation of amide or carboxyl. The peak at 1000–1270 cm^−1^ was originated from stretching vibrations of C-O, implying the existence of ether group or hydroxyl. Furthermore, the peak at 764 cm^−1^ was ascribed to the out-of-plane bending vibration of O-H or C-H demonstrated the ring-shaped conjugated structures in rCDs.

To verify the inference described above, the survey X-ray photoelectron spectroscopy (XPS) was employed. Figure 3D clearly shows the binding energy peaks of C 1 s, N 1s, and O 1s, while the peaks of sodium of Na 1s, Na 2s, Na 2p, and Na KL1 were ascribed to the addition of NaOH in the synthesis of rCDs. The high-resolution XPS showed the three peaks of C 1s (Figure 3E), corresponding to 284.8 eV (C=C/C-C), 285.9 eV (C-N/C-O/C=N), and 288.3 eV(O=C-O). Two fitting peaks of N 1s at 399.9 eV and 397.7 eV were attributed to the pyridinic N and pyrrolic N, respectively (Figure 3F). Figure 3G confirms the presence of C=O (531.5 eV) bonds, O=C-O group (533.5 eV), and the C-O of the aromatic nucleus (535.5 eV). In general, the results of XPS showed good agreement with the generation of the aromatic group from FTIR analysis.

### 3.4. Development of FIA

**Figure 4:**
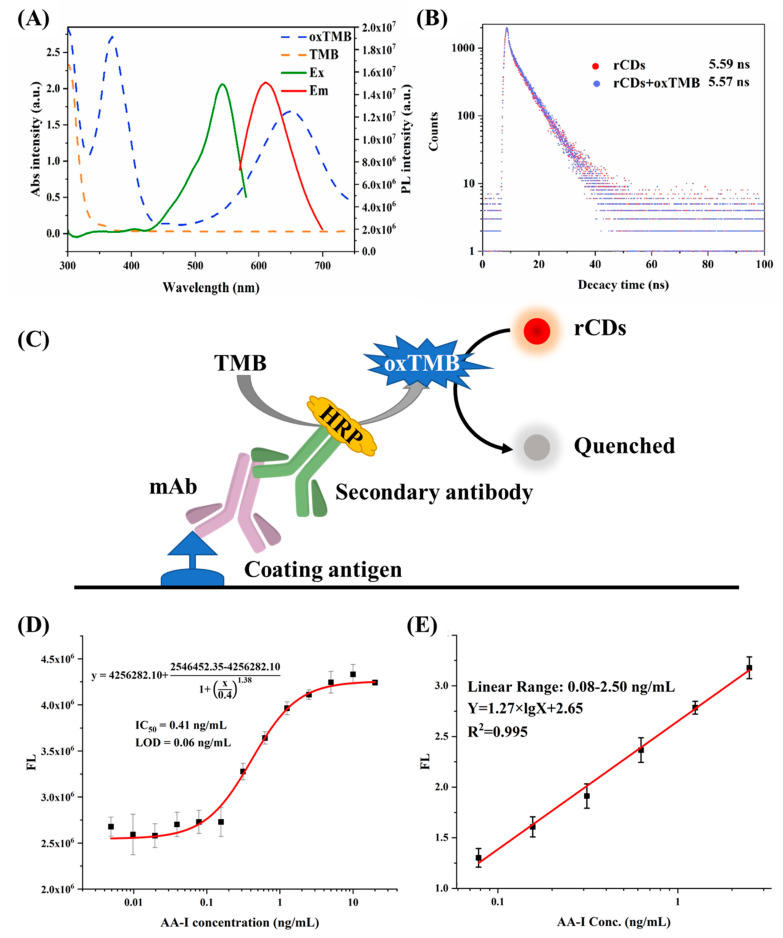
(**A**) The spectrum characterization of rCDs, TMB, and oxTMB. (**B**) The lifetime analysis of rCDs with and without oxTMB. (**C**) Strategic schema of development of FIA. (**D**) Calibration curve and (**E**) linear range of FIA. Acronym: TMB: 3,3′′,5,5′′-tetramethylbenzidine dihydrochloride; oxTMB: oxidized TMB; Em: emission; Ex: excitation.

The spectral characteristic of rCDs, TMB, and oxTMB was investigated to assess the feasibility of FIA development. Figure 4A shows the Em wavelength of rCDs at 620 nm with the Ex wavelength at 540 nm. Compared with TMB, the oxTMB exhibited an obvious absorbance peak at 650 nm, which overlaps the Em of rCDs, thereby quenching the fluorescent signal of rCDs. Therefore, the presence of HRP can catalyze TMB into oxTMB to quench rCDs; otherwise, the fluorescent signal was a turn-on. The fluorescence lifetime of rCDs was investigated. In the presence of oxTMB, the fluorescence lifetime of rCDs (5.57 ns) showed no obvious difference to rCDs without oxTMB (5.59 ns), indicating the inner-filter effect caused the quenching (Figure 4B). The fluorescent intensity of rCDs with various pH values was also studied, and the highest intensity was observed for the pH value of 11 (Figure S4). Therefore, rCDs were diluted by NaOH (pH 11) to adjust the pH value before adding to microplates.

Based on the quenching mechanism, an rCDs-based FIA was developed, strategy diagram is shown in Figure 4C. In the absence of AA-I, the mAb was bound to coating antigen, resulting in the generation of oxTMB, leading to the quenching of rCDs. In contrast, the presence of AA-I inhibited the binding of mAb to coating antigen, recovering the fluorescent signal of rCDs. Hence AA-I concentration regulated the fluorescence response. Based on the above optimized conditions, a fluorescent calibration curve against AA-I showed the IC_50_ of 0.41 ng/mL (Figure 4D), the LOD of 0.06 ng/mL, and a linear range from 0.08 to 2.5 ng/mL. Compared with other publications for AA-I analysis, the developed FIA showed higher sensitivity (LOD) (Table 3), simplicity, and high efficiency without a complicated procedure for sensitivity improvement, which makes the FIA suitable for sample screening. The downsides are that the FIA still requires at least approximately 90 min to detect AA-I, and the microplate reader requirement limits the on-site detection of FIA. To overcome these shortcomings, handheld reader-based lateral flow immunoassays will be developed in future work for more rapid and on-site detection of AA-I.

### 3.5. Recovery Test

Three levels of AA-I were spiked to drug homologous and foods then analyzed by CLEIA and FIA. The results were verified by UPLC-QQQ-MS/MS, which is shown in Table 4. The recoveries of CLEIA and FIA were ranged between 83–119% and 86–118.4% with a coefficient of variance (CV) ranging from 3.8% to 13.3% and 5% to 14.4%, respectively. The UPLC-QQQ-MS/MS showed the average recoveries from 85.1% to 108.1%, with CVs from 1.2% to 7.5%. The developed CLEIA and FIA showed good agreement to standard instrument methods, demonstrating good accuracy and practicability for AA-I detection.

^1^ SD, standard deviation; ^2^ CV, coefficient of variance.

## 4. Conclusions

In this study, the AA-I was conjugated to a carrier protein to prepare artificial antigen, and a sensitive anti-AA-I mAb was generated after animal immunization and hybridoma screening. The obtained mAb was further used to develop CLEIA for the detection of AA-I. Since products containing AAs are prohibited in most countries, high sensitivity methods are needed to screen out positive samples, but the sensitivity of CLEIA was still not satisfying. Therefore, CDs with red emission were synthesized and employed to develop FIA, which exhibited a five-fold improvement in sensitivity than CLEIA. The accuracy and practicability of CLEIA and FIA were verified by the standard instrument method; they were sensitive, rapid, and easy to use, making them effective tools for screening AA-I in related products.

## Figures and Tables

**Table 1:** Characterization of mouse antiserum against AA-I (*n* = 3).

Mouse	Immunogen	Coating Antigen ^1^	Titer	IC_50_ (ng/mL)
1	AA-I-BSA	AA-I-OVA	16K	120.3 ± 13.0
2	AA-I-BSA	AA-I-OVA	32K	148.8 ± 11.8
3	AA-I-BSA	AA-I-OVA	16K	156.1 ± 16.2
4	AA-I-KLH	AA-I-OVA	64K	27.3 ± 4.3
5	AA-I-KLH	AA-I-OVA	64K	56.6 ± 7.7
6	AA-I-KLH	AA-I-OVA	32K	35.8 ± 3.6

^1^ The concentration of coating antigen was 1 μg/mL.

**Table 2:** The specific test of anti-AA-I mAb.

Compounds	Structure	IC_50_ (ng/mL)	CR ^1^ (%)
AA-I	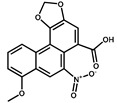	1.8	100
AA-II	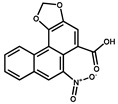	2.1	86
AA-III	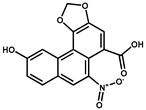	120.0	1.5
AA-IV	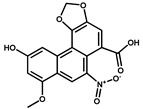	450.0	0.4
Abietic acid	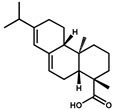	>1000	<0.1
Asarinin	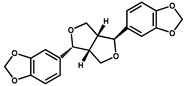	>1000	<0.1
Colchicine	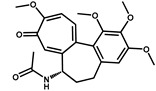	>1000	<0.1
Methyleugenol	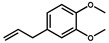	>1000	<0.1
Ephedrine hydrochloride	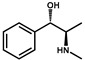	>1000	<0.1

^1^ CR(%) = [IC_50_ (AA-I)/IC_50_ (analogues)] × 100%.

**Table 3:** The comparison of immunoassay for AAs.

Method	IC_50_ (ng/mL)	Linear Range (ng/mL)	LOD (ng/mL)	Reference
icELISA	1.2	ND ^1^	0.1	1
Injection analysis chemiluminescence	ND ^1^	10–20000	3	4
CLEIA	2.4	0.2–3.1	0.1	This work
FIA	0.41	0.08–2.50	0.06	This work

^1^ ND, no data.

**Table 4:** Recovery test for CLEIA, FIA, and UPLC-QQQ-MS/MS (*n* = 3).

Sample No.	Spiked (ng/g)	CLEIA			FIA			UPLC-QQQ-MS/MS		
		Measured (ng/mL) (Mean ± SD ^1^)	Recovery (%)	CV ^2^ (%)	Measured (ng/mL) (Mean ± SD)	Recovery (%)	CV (%)	Measured (ng/mL) (Mean ± SD)	Recovery (%)	CV (%)
Pleurotus ostreatus	600	511.4 ± 41.4	86.7	7.7	655.2 ± 92.4	109.2	14.1	648.3 ± 11.9	108.1	1.8
	300	165.3 ± 21.9	83.0	13.3	355.2 ± 55.2	118.4	15.5	303.7 ± 12.9	101.2	4.2
	150	135.1 ± 14	90.0	12.8	129 ± 18	86	14.0	127.7 ± 9.6	85.1	7.5
Slimming capsule	600	520.1 ± 11.3	86.7	10.4	642 ± 92.4	107	14.4	571.3 ± 12.9	95.2	2.3
	300	237.9 ± 30.4	119.0	12.6	306 ± 28.8	102	9.4	313.7 ± 16.5	104.6	5.3
	150	116 ± 41.1	116.0	36.2	147 ± 21	98	14.3	136 ± 5.6	90.7	4.1
Slimming tea	600	517.4 ± 11.8	86.7	3.8	678 ± 62.4	113	9.2	644.3 ± 7.5	107.4	1.2
	300	204.9 ± 45.7	102.0	22.5	312 ± 15.6	104	5.0	305.3 ± 11.9	101.8	3.9
	150	114.3 ± 13.3	114.0	12.3	138 ± 9	92	6.5	155.3 ± 6.5	103.5	4.2

## Data Availability

The datasets used and analyzed during the current study are available from the corresponding author on request.

## Supplementary Materials

The following are available online at https://www.mdpi.com/article/10.3390/foods10112647/s1. Figure S1: Ethical review of animal experiments. Figure S2: Ultraviolet scanning of synthesized antigens of AA-I (A) Ultraviolet spectrum of AA-I, KLH and AA-I-KLH; (B) AA-I, BSA and AA-I-BSA; (C) AA-I, OVA and AA-I-OVA. Figure S3: The optimizing of (A,B) ionic strength; (C,D) pH value; (E,F) Tween-20 concentration; (G, H) methanol concentration. Figure S4: The optimizing of pH for rCDs. Table S1: Parameters of UPLC-QQQ-MS/MS. Table S2: Cell lines evaluation (*n* = 3). Table S3: Optimization of coating antigen concentration (*n* = 3).
