# Supplementary material for: Preparation of Anti-Aristolochic Acid I Monoclonal Antibody and Development of Chemiluminescent Immunoassay and Carbon Dot-Based Fluoroimmunoassay for Sensitive Detection of Aristolochic Acid I
Source: Foods. 2021 Nov 1;10(11):2647. doi: 10.3390/foods10112647 (PMC8622072; doi:10.3390/foods10112647)
Supplement: Supplementary file 1 [file foods-10-02647-s001.zip › foods-1417324-Supplementary materials pdf.pdf]

Supplementary Materials

Preparation of Anti-Aristolochic Acid I Monoclonal Antibody and Development of Chemiluminescent Immunoassay and Carbon Dot-Based Fluoroimmunoassay for Sensitive Detection of Aristolochic Acid I

Ai-Fen Ou[1,2], Zi-Jian Chen[3], Yi-Feng Zhang[3], Qi-Yi He[1], Zhen-Lin Xu[3], Su-Qing Zhao[1,*]

1 Department of Pharmaceutical Engineering, School of Biomedical and Pharmaceutical Sciences, Guangdong University of Technology, Guangzhou 510006, China; ouaifen@gcp.edu.cn (A.-F.O.); chesto36@163.com (Q.-Y.H.)

2 Department of Food, Guangzhou City Polytechnic, Guangzhou 510006, China

3 Guangdong Provincial Key Laboratory of Food Quality and Safety/Guangdong Laboratory of Lingnan Modern Agriculture, South China Agricultural University, Guangzhou 510642, China; guangdongchenzj@163.com (Z.-J.C.); zhyifengscau@163.com (Y.-F.Z.); jallent@163.com (Z.-L.X.)

* Correspondence: sqzhao@gdut.edu.cn

Synthesis of immunogens and coating antigens. AA-I was conjugated to BSA or KLH as immunogen and was conjugated to OVA for coating antigens using the active ester method. Briefly, AA-I (0.025 mmol), EDC (0.0375 mmol) and NHS (0.0375 mmol) were dissolved by 0.6 mL of DMF and stirred at for 4 h. Then the mixture was added to 50 mg protein (dissolved in 5 mL 50 mM pH 9.6 carbonate Buffer) and adjusted pH to 9.6 by 3 M NaOH. The solution and stirred overnight. Then the solution was dialyzed by 0.01 M PBS (5 L) for 3 days, and finally stored at -20 ºC until use. UV-vis spectral data was used to confirm the structure of the final conjugates (Figure S2).

Synthesis of rCDs preparation. One gram of citric acid and 2 g of urea were dissolved in 10 mL DMF and then the mixture was heated to 160 °C for 6 hours. The obtained dark red product was mixed with 20 mL NaOH (50 mg/mL), and then centrifuged 10 min at 14000 rpm (18407×g). The precipitate was dissolved with 10 mL ultrapure water and centrifuged again to remove residual salts. Finally, the rCDs was resuspended with 10 mL ultrapure water.

華南農業大學
South China Agricultural University

Results of ethical review of animal experiments

No：2019054

Experiment Item	The preparation of monoclonal antibody for pesticides and their metabolite			
Application number	2019B054			
Comments on conservation of experimental animals	All the experimental mice used in this experiment came from experimental animal centre legal license. The type, quantity and grouping of mice were conformed to the 3R principle.			
Comments on welfare assessment of experimental animals	This experiment was carried out in a laboratory with a license for experiment animal, which was conformed to the welfare principle.			
Comments on ethical and moral	The animals were euthanized after the experiment.			
Comments on comprehensive scientific evaluation	This experimental study has scientific significance.			
Time of experiment animal type and quantity	Date: 2019-05-15 to 2019-07-30.<br>Experimental animal: SPF BAL B/c female mice.<br>Quantity: 70.			
Comments of ethical reviewer	Agree			
	Reviewer	Zhonghua Liu	Review Date	2019-05-08
Comments of ethical reviewer	Agree			
	Reviewer	Wei Huang	Review Date	2019-05-08
Final comments of director (or deputy director)	Agree			
	Reviewer	Ming Liao	Review Date	2019-05-08

Experimental Animal Ethics Committee of South China Agricultural University

Date 2019-05-08

Figure S1. Ethical review of animal experiments

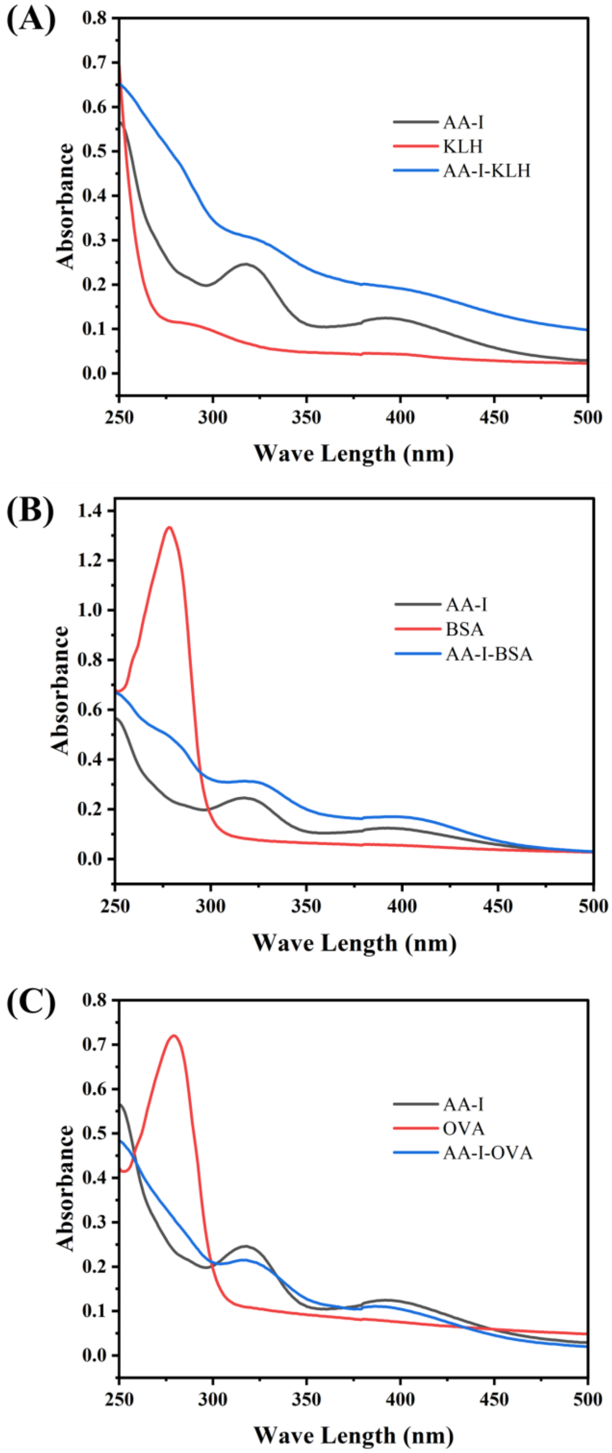

Figure S2. Ultraviolet scanning of synthesized antigens of AA-I (A) Ultraviolet spectrum of AA-I, KLH and AA-I-KLH; (B) AA-I, BSA and AA-I-BSA; (C) AA-I, OVA and AA-I-OVA.

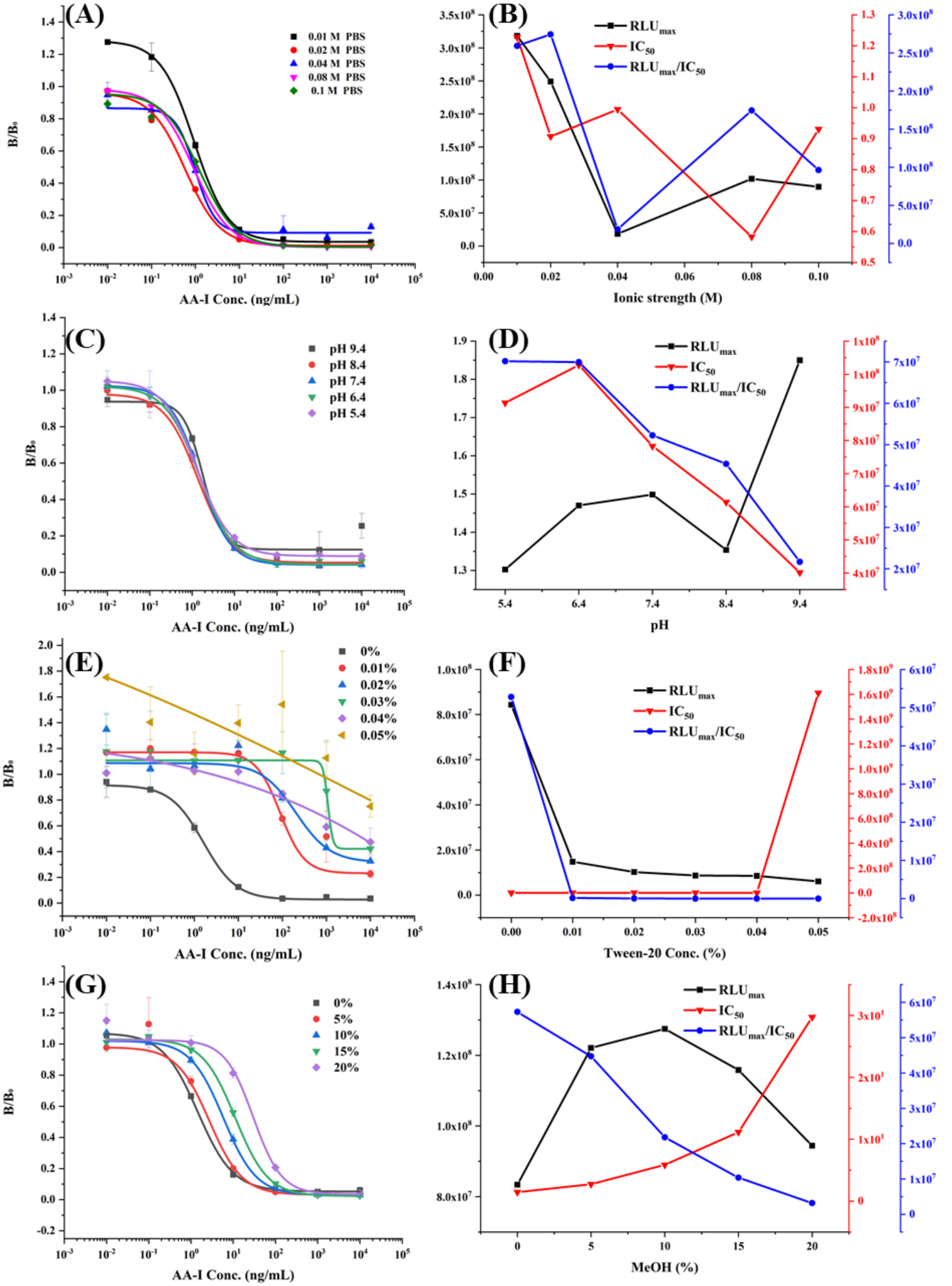

Figure S3. The optimizing of (A, B) Ionic strength; (C, D) pH value; (E, F) Tween-20 concentration; (G, H) Methanol concentration.

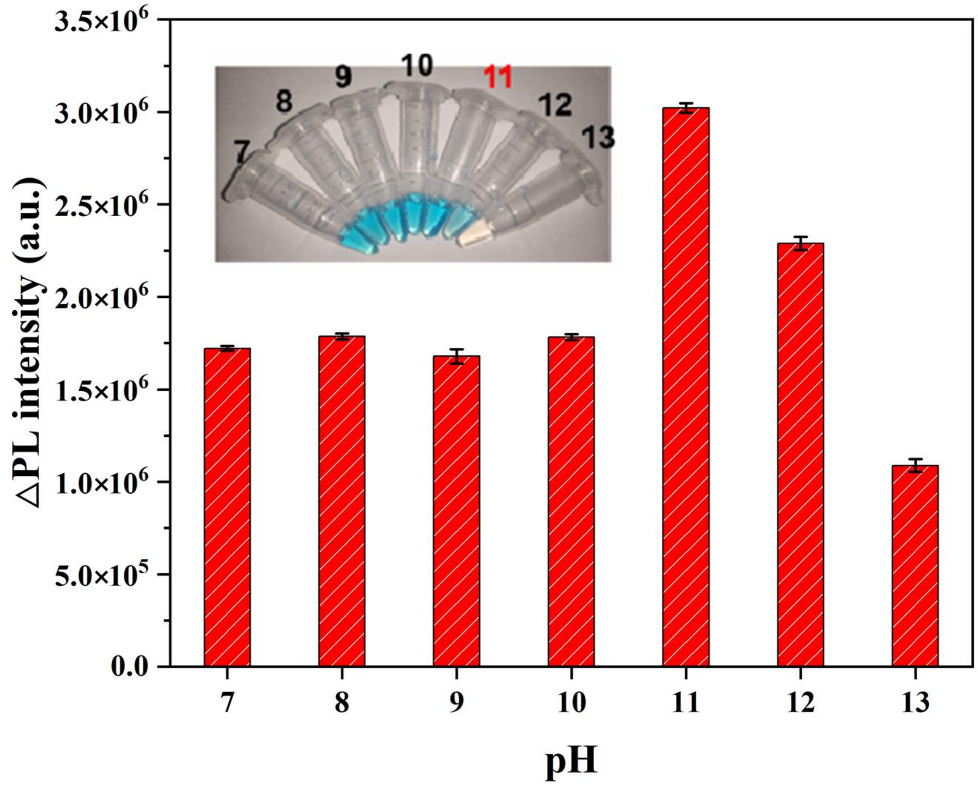

Figure S4. The optimizing of pH for rCDs.

Table S1 Parameters of UPLC-QQQ-MS/MS

Item	Parameters
Ion source	Electrospray ionization (ESI+)
Detection mode	multiple reaction monitoring (MRM)
Scanning mode	Positron mode
Curtain Gas	30μL/min
ionizing voltage	5500V
Ion source temperature	550 ℃
Retention time	4.26 min
Parent ion (*m/z*)	359.100 $[M+H]^+$
Daughter ion (*m/z*)	298.100[1]
Declustering potential	65V
Collision Energy	16eV

[1]Quantification ion pair

Table S2 Cell lines evaluation (n=3)

Cell clonal image	Cell clonal number	IC_{50} (ng/mL)[1]
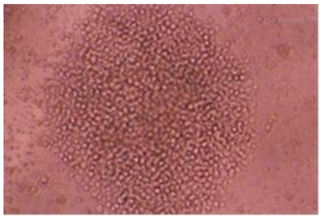	3A3	11.3±1.7
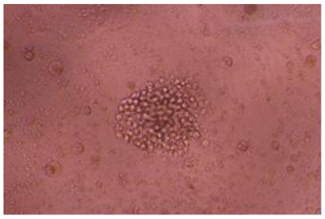	5C12	19.2±1.4
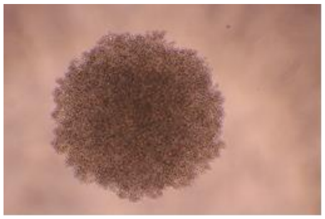	1H7	32.5±3.2
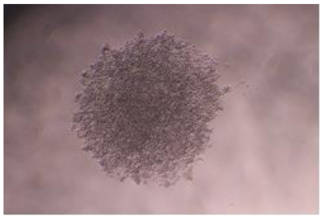	1B4	22.7±4
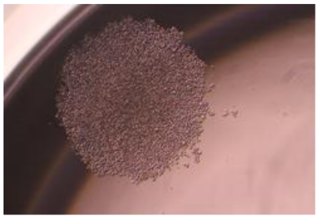	7G6	30.2±4.3
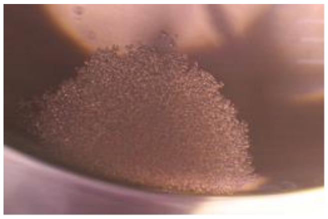	4B1	25.6±3.7

[1] The concentration of coating antigen was 1 μg/mL.

Table S3 Optimization of coating antigen concentration (n=3)

Coating antigen concentration (μg/mL)	mAb concentration (ng/mL)	RLU_0 (mean±SD[1])	RLU[2]	Inhibition[3] (%)
2	1000	384970580±5310223	388682380±13355825	-1
	500	154411557±11373230	151221537±1212134	2.1
	250	458939515±57755509	69235764±697191	84.9[4]
	125	198831376±22622577	32214033±2866292	83.8
1	1000	268208914±25985236	47788119±637271	82.2
	500	128413594±464195	20315647±1694822	84.2
	250	61572049±8845561	20628542±2588584	66.5
	125	37050641±7523820	16283822±1275431	56
0.5	1000	221422515±18848038	35112256±225114	84.1
	500	137213144±6674755	24112574±2570426	82.4
	250	78199741±21590849	22024911±9894031	71.8
	125	29837089±5007276	14996433±1773348	49.7
0.25	1000	41759552±8255673	7287115±1253298	82.5
	500	21875743±629344	5264211±974094	75.9
	250	10716646±119017	6962599±1272932	35
	125	5020236±465055	4306175±807786	14.2

[1] SD, standard deviation;

[2] RLU was measure with 1 ng/mL of AA-I;

[3] Inhibition = $(1-RLU/RLU_0) \times 100\%$;

[4] The coating antigen concentration performed the highest inhibition was chosen as the optimized condition.
